# Circular Penile Skin Fasciocutaneous Ventral Onlay Flap Urethroplasty as an Alternative to Dorsal Onlay Buccal Mucosal Graft Urethroplasty in Complex Long-Segment Urethral Stricture: A Retrospective Study

**DOI:** 10.7759/cureus.45084

**Published:** 2023-09-12

**Authors:** Rana P Singh, Arshad Jamal

**Affiliations:** 1 Department of Urology, Rajendra Institute of Medical Sciences, Ranchi, IND

**Keywords:** urethroplasty, urethra, penile, flap, buccal

## Abstract

Background

A urethral stricture is the narrowing of the urethra that results in symptoms of obstruction. It can appear anywhere along the male urethra's length and has a variety of causes. The circular penile fasciocutaneous flap is employed in the successful single-stage reconstruction of long-segment complex anterior urethral strictures especially when the buccal mucosa is unavailable due to various reasons. The study has tried to identify a surgical technique that is more beneficial for the treatment of urethral strictures.

Objective

The objective of this research was to evaluate the outcomes of circular penile skin fasciocutaneous ventral onlay flap urethroplasty (group A) and the outcomes of dorsal onlay buccal mucosal graft urethroplasty (group B) in the management of complex long-segment penile urethral stricture.

Methods

In this retrospective study between December 2012 and December 2022, 60 patients with long-segment complex penile urethral stricture who underwent urethroplasty at our center were evaluated. Patients were divided into two groups according to the flap used (dorsal onlay buccal mucosal graft urethroplasty was used in 30 patients (group B), and circular penile fasciocutaneous flap (single stage) was used in 30 patients (group A)). The success rate and the mean peak flow rate were also calculated post-operation to identify the effectiveness of the surgical procedure used for urethral strictures.

Results

The study consisted of 60 patients in total. Group A's mean age was determined to be 51.2±16.2 years, whereas group B's mean age was determined to be 40.7±16.8 years. Preoperatively, the median urethral stricture length was 69 mm in group A (range: 20-100 mm) and 56 mm in group B (range: 30-110 mm). The intraoperative median length of the urethral stricture was 82 mm in group A (range: 20-120 mm) and 65 mm in group B (range: 40-140 mm). The mean peak flow rate was 30.9±6.8 mL/s in group A compared to 18.1±4.9 mL/s in group B. The success rate for group A was 89.7%, while the success rate for group B was 75.9%.

Conclusion

For complex long-segment urethral strictures, circular penile skin fasciocutaneous ventral onlay flap urethroplasty has a higher rate of success and fewer complications than dorsal onlay buccal mucosal graft urethroplasty. Along with success rate, it has a better mean peak flow rate and lower complications.

## Introduction

Urethral strictures are a diverse disease. A successful repair frequently necessitates a variety of surgical procedures. The scarring of subepithelial tissue enclosed in the corpus spongiosum results in male anterior urethral strictures, which constrict the urethral lumen and decrease urine flow. Its surgical care is a difficult issue that has undergone significant change over the past few decades. Options for treatment include urethrotomy, dilatation, and reconstructive surgical procedures [1]. The ideal technique for treating urethral stricture is tissue transfer using a flap or a graft. The graft can be retrieved from the buccal, intestinal mucosa, or even penile skin. Graft or flap urethroplasty can be employed when anastomotic urethroplasty is not an option. The issue is that no one technique is appropriate for all circumstances, and the urologist treating this disease should be familiar with the majority of them [2].

Although numerous research studies have looked at the use of urethroplasty to treat short-segment anterior urethral stricture, very few have explored the challenging issue of long-segment stricture [3]. Nowadays, the gold standard for the treatment of anterior urethral stricture is substitution urethroplasty. This technique is employed when the strictures do not qualify to be treated with primary anastomosis or excision. The buccal mucosa, which is removed from the inner cheek, works well as a replacement for urethroplasty material. However, there is a need to evaluate the effectiveness of penile skin flap in the management of urethral strictures particularly in the long-segment complex urethral strictures [4]. Furthermore, the scientific literature also lacks robust evidence on the definition of long-segment urethral stricture; some studies used a cutoff of 8 or 9 cm, while others characterized it as having more than one stricture site [5,6].

This retrospective study aimed at comparing the effectiveness of circular penile skin fasciocutaneous ventral onlay flap urethroplasty (single stage) with dorsal onlay buccal mucosal graft urethroplasty for the management of complex long-segment urethral strictures.

## Materials and methods

This retrospective observational study has included 60 patients. A total of 30 patients underwent circular penile skin fasciocutaneous ventral onlay flap urethroplasty (group A), and another 30 patients underwent dorsal onlay buccal mucosal graft urethroplasty (group B) at Rajendra Institute of Medical Sciences (RIMS), Ranchi, between December 2012 and December 2022 for complex long-segment urethral stricture (≥8 cm). Patients with a history of urethroplasty or hypospadias repair were excluded from the study, as were those with unhealthy penile skin, balanitis xerotica obliterans stricture, lichen sclerosus, a very tiny urethra, or both. Data regarding patient details including clinical history, urine analysis, urine culture, pelvic abdominal ultrasonography for estimating post-void residual urine, detailed surgical histories, uroflowmetry, retrograde urethrography, and voiding cystourethrography were collected.

Surgical intervention

The patients included in the study either underwent dorsal onlay buccal mucosal graft urethroplasty or circular penile skin fasciocutaneous ventral onlay flap urethroplasty. The surgical team and the surgeon's preferences, as well as his area of expertise, all had a role in determining the type of procedure being employed for the patient.

Ventral onlay flap urethroplasty was conducted following McAninch et al. [7]. Stricturotomy was carried out up to the healthy, ventrally visible urethral tissue. After determining the extent of the urethral stricture, a circular penile skin fasciocutaneous flap was created. The distal circular penile flap was harvested by employing a sub-coronal circumferential incision of approximately 15-20 mm. The extent of the estimated incision was determined depending on the amount of urethral plate available. After dissecting the subdermal skin and the Dartos fascia, a plane was formed between the superficial lamina of Buck's fascia and the Dartos fascia, which approached the penis root. Both the vascular pedicle and the flap were incised longitudinally to form a long strip of skin. Following that, a new urethra was formed over the urethral silicone catheter and sealed using a tension-free, watertight vicryl suture, and glanuloplasty was done. The accessible surrounding fascia was used to cover the simultaneous anastomosis. Furthermore, the penis was put in a stretched position to avoid redundancy.

Dorsal onlay buccal mucosal graft urethroplasty was conducted as described by Barbagli et al. [8]. The patients were positioned in the lithotomy position. The entire procedure was conducted under general anesthesia, based on where the stricture was situated. The patient was administered general anesthesia, and a nasal intubation was also employed for performing the buccal mucosal transplantation. The buccal mucosal grafts were obtained from the following locations: inner cheeks, lower lips, or both locations. The buccal grafts were obtained based on the length of the stricture. The urethra was rotated 180 degrees away from its dorsal surface after it was separated from the corpora cavernosa. To determine the length of the stricture, the urethra was exposed to healthy urethral mucosa after instilling methylene blue into the urethra. To cover the corpora cavernosa, the graft was fenestrated, defatted, and constantly sewn over a silicone catheter with vicryl stitches. The most proximal and distal portions of the flap were secured to the normal urethra using interrupted 4-0 vicryl, and flap suturing was completed. The surgeries were carried out by two teams: one for urethroplasty and another for buccal graft harvesting.

Cephalosporins (1 gm), particularly the third generation, were incorporated 24 hours before the surgery was done.

Both groups received broad-spectrum antibiotics beginning an hour before surgery and continuing for five days afterward. After that, an oral antibiotic was given until the surgically implanted catheter was removed.

Follow-up

The catheter was removed four weeks after the surgery, and no extravasation was detected. However, patients with evidence of wound infection had their catheter in place for an extra one to two weeks.

After three and six months, or earlier if obstructive symptoms were visible, uroflowmetry and retrograde urethrography were performed. Following that, symptomatic evaluation and uroflowmetry were performed every six months. We conducted urethrography only when symptoms or uroflowmetry indicated a recurrent stricture. A peak flow rate of more than 15 mL/s [9] and the absence of any postoperative instrumentation requirements were considered the conditions for successful reconstruction.

The study was carried out following the Helsinki Declaration and was approved by the Institutional Ethics Committee of RIMS, Ranchi (approval letter number: 116, protocol submission number: R 14/23).

## Results

In our study, a total of 60 patients with complex long-segment penile urethral stricture were enrolled. The mean age was 51.2±16.2 years in the group that received ventral onlay flap urethroplasty and 40.7±16.8 years in the group that underwent dorsal onlay buccal mucosal graft urethroplasty. Preoperatively, utilizing combined ascending and voiding urethrography, the median urethral stricture length was 69 mm in group A (range: 20-100 mm) and 56 mm in group B (range: 30-110 mm). When the stricture length was measured intraoperatively, there was a variance in the stricture length. The intraoperative median length of the urethral stricture was 82 mm in group A (range: 20-120 mm) and 65 mm in group B (range: 40-140 mm) (Table 1).

**Table 1:** Patient data collected preoperatively and intraoperatively SD: standard deviation

Preoperative data		
	Group A	Group B
Etiology		
Inflammatory	8 (26%)	20 (66.6%)
Post-catheterization	5 (16.6%)	17 (56.6%)
Idiopathic	23 (76.6%)	2 (6.6%)
Iatrogenic trauma	4 (13.3%)	1 (3.3%)
Length of urethral stricture in mm		
Range	20-100	30-110
Median	69	56
Intraoperative data		
Length of urethral stricture in mm		
Range	20-120	40-140
Median	82	65
Operative time in minutes		
Mean ± SD	184.21±29.2	187.52 ±29.6

Depending on the location and length of the stricture, group A used local circular penile flaps. In group B, dorsal onlay buccal graft urethroplasty was performed on all patients.

During the follow-up period of three months (before catheter removal), patients underwent follow-up pericatheter urethrogram; no patient had extravasation of contrast. After three months, patients were checked again with uroflowmetry, retrograde urethrography, and micturating cystourethrography.

The mean peak flow rate in group A was 25.96±6.8 mL/s, while in group B, it was 18.14±9.9 mL/s. One (3.3%) patient in group A and three (10.1%) patients in group B had a peak flow rate of less than 15 mL/s after three months of follow-up. At the conclusion of a year of follow-up, three more patients in group B had strictures with peak flow rates of less than 15 mL/s. Following the results of the wound swab culture and sensitivity tests, two (6.6%) patients in group A and six (20%) patients in group B developed wound infections, which were treated with regular dressing and antibiotics, respectively.

One (3.3%) patient in group A experienced meatus regression to the sub-coronal location. The patient was offered surgical repair, but he declined since he was satisfied with the results of the surgery. Furthermore, no patients in group A presented with ring urethral stricture. Three (10%) patients in group B experienced minor distal penile fistula. Three more patients in group B experienced postoperative minor penile chordee. All patients in group B who had oral problems experienced some degree of minor pain at the donor site. Five patients experienced moderate mouth-opening restrictions. In three cases, mouth numbness and salivary disruption happened.

In our investigation, the urethral stricture that developed after surgery was regarded as a sign that the procedure had failed. Group A had a success rate of 89.7%, and Group B had a success rate of 75.9% (Table 2).

**Table 2:** Patient data collected postoperatively SD: standard deviation

	Group A	Group B
Uroflow Qmax (mL/s)		
Mean±SD	25.9±6.8	18.1±4.9
Less than 15	3.3%	10%
More than 15	96.7%	90%
Presence of early complication		
No	93.4%	80%
Yes (in case of wound infection)	6.6%	20%
Success rates	89.7%	75.9%

## Discussion

Since the Orandi technique was introduced in 1968, urethroplasty with a penile skin flap has become the standard procedure [10]. McAninch presented a circular fasciocutaneous flap to replace Orandi's longitudinal penile skin flap for the treatment of a penile urethral stricture in 1993. Because penile skin is a flexible, hairless tissue with a strong circulatory supply from the surrounding tissues, it is a successful procedure with outstanding cosmetic and functional results [11].

It is still argued whether a flap or graft is best suited for replacing the urethra [12]. The employment of one technique over another, however, is not explicitly supported by the present literature [13]. Due to its beneficial histological features and high success rate, the buccal mucosa became the norm for repairing urethral strictures in the late 1990s. However, there are several drawbacks to take into account. For oral graft harvest, it is necessary to have a second operating room and more skilled medical and/or surgical staff. Additionally, several donor site-related problems, such as oral pain, mouth tightness, and changes in saliva production, have been documented [14]. In our study, multiple oral problems were reported by patients in group B, which included pain at the donor site, mouth-opening restrictions, mouth numbness, and salivary disruption.

Stein et al. have reported that the mean age of onset of stricture diseases in males is 41.4 years [15]. Furthermore, Santucci et al. have also concluded that the incidence of urethral strictures increases markedly after 55 years of age [16]. A similar pattern was obtained in our study where the mean age of patients included in both groups ranged from 40 to 50 years.

All reconstructive urologists are aware that penile urethroplasty and bulbar urethroplasty employing single-stage flap or graft procedures differ in the surgical steps, potential risks, and outcomes. It is also widely recognized that the duration and etiology of the stricture have an impact on how well urethroplasty treatments work. For these reasons, cases of lichen sclerosus, extremely narrow or obliterated urethras, and people who had undergone urethroplasty or had their hypospadias repaired in the past were not included in this retrospective study. Furthermore, in this study, the stricture length was assessed both intraoperatively and postoperatively to ensure the accuracy of the values observed and avoid discrepancies. The intraoperative median length of the urethral stricture was 82 mm in group A (range: 20-120 mm) and 65 mm in group B (range: 40-140 mm). However, a discrepancy of approximately 10%-20% was observed between the preoperative and intraoperative length as the length calculated intraoperatively included up to the healthy urethral tissue.

The intraoperative measurement of stricture length is more reliable and accurate than the preoperative measurements. Gupta et al. have reported that sono-urethrography gives a much more accurate length of the urethral stricture than retro-urethrography [17]. Furthermore, sono-urethrography gives measurements similar to that found intraoperatively. However, we have included only retro-urethrography for the measurement of stricture length, and therefore, the discrepancy in the measurement of urethral stricture is a limitation of this study. As a result, we believe that retro-urethrography is a useful method for identifying the precise position and length of the stricture. However, data must be thoroughly analyzed, and the reconstructive urologist must be knowledgeable and well-equipped to handle any unexpected events that may arise during surgery.

The initial study by McAninch and Morey found that a circular penile fasciocutaneous flap had a 79% overall success rate with a mean follow-up of 41 months [7]. Whitson et al. have reported that a penile circular fasciocutaneous flap for one-stage repair has high long-term efficacy for the management of anterior urethral strictures [18]. According to Schwentner et al., single-stage penile skin fasciocutaneous ventral onlay flap urethroplasty had a long-term success rate of 90% with a mean follow-up period of 96.7 months [19]. Similar trends were observed in our investigation, which determined that group A had an overall success rate of 89.7%.

According to Dubey et al., pendulous, bulbar, and bulbo-pendulous strictures, mean stricture length, and median follow-up were all comparable between buccal mucosa dorsal onlay and penile skin flap urethroplasty [20]. In addition, one patient in the penile flap group required skin grafting due to significant skin loss, and two patients developed twisting of the penile tissue. However, in our study, only one patient experienced meatus regression to the sub-coronal location, and no patients experienced ring urethral stricture. Very few cases of penile skin necrosis were documented. It happens when the subdermal plexus's vascular supply is impaired. Although it occurs less frequently in the hands of competent medical professionals, this is an inherent drawback of any pedicle penile skin flap [21]. There were no cases of penile skin necrosis reported in our study.

It is still up for debate whether ventral or dorsal transplant placement is better. Some researchers believe that ventral placement of the flap or graft may result in urethral diverticula, succulations with post-void dribbling, and ejaculatory failure [21]. Others, however, have reported using this procedure with satisfactory long-term stricture-free results in comparison to dorsal onlay buccal mucosal graft [22]. In our study, it was found that the ventral method offered a better advantage, and the outcomes of this technique are encouraging.

Since we have been using this method at our institution for a long time, we believe that all urologists can safely and easily harvest penile skin because it does not require any special training in oral surgery or an understanding of the architecture of the mouth. Based on the previously published research, there is clear evidence that penile skin flap replacement urethroplasty results in positive patient outcomes.

We recognize that the relatively brief follow-up in our study reduces the number of potential post-complex surgery problems, such as diverticula development, late stricture recurrence, and micturition dysfunction. We also acknowledge the absence of data on sexual dysfunction before and during treatment in our study. Furthermore, in our study, the choice of suture, bandage, catheter used, and further details could not be reproduced as it was a retrospective study. Hence, we acknowledge these limitations in our study. Neither penile Doppler results nor erectile function scores were evaluated in our patients. Furthermore, large prospective series with extensive follow-ups are required for better outcomes.

## Conclusions

The effects of this obstruction related to urethral stricture can significantly reduce the patient's quality of life. Therefore, it is important to identify techniques with better success rates and lower complications. A lengthy and difficult anterior urethral stricture may be successfully treated with substitution urethroplasty employing a penile skin flap. It yields encouraging aesthetic and practical outcomes.
